# Inter-Vendor Variability of Perfusion Parameters Derived from Dynamic Contrast-Enhanced MRI in Patients with Prostate Cancer

**DOI:** 10.3390/tomography12070091

**Published:** 2026-06-23

**Authors:** Mingyu Kim, Seung Ho Kim, Joo Yeon Kim

**Affiliations:** 1 Department of Radiology, Haeundae Paik Hospital, Inje University College of Medicine, Busan 48108, Republic of Korea; 2 Department of Pathology, Haeundae Paik Hospital, Inje University College of Medicine, Busan 48108, Republic of Korea

**Keywords:** perfusion, dynamic contrast-enhanced (DCE), magnetic resonance imaging (MRI), prostate cancer (PCa), Gleason score (GS)

## Abstract

**Purpose**: To investigate the agreement on perfusion parameters derived from two different commercially available solutions for dynamic contrast-enhanced magnetic resonance imaging (DCE-MRI) in patients with prostate cancer (PCa). **Methods:** A total of 50 patients (mean age, 71.6; range 56–86) who had undergone radical prostatectomy between December 2021 and September 2022 were included in this retrospective study. All patients had undergone DCE-MRI on a single 3T-MR scanner. Tumor segmentation on MR images was performed by two radiologists in consensus after radiologic-pathologic correlation using topographic maps as a reference standard. Subsequently, four perfusion parameters were calculated by dedicated commercially available solutions from two different vendors. Both solutions adopted a population-based arterial input function and an extended Tofts model as the pharmacokinetic model. The perfusion parameters were as follows; volume transfer constant (K^trans^), rate constant (k_ep_), volume fraction of extravascular extracellular space (v_e_), and volume fraction of plasma (v_p_). The differences between paired measurements were compared by Bland–Altman analyses and the reproducibility was evaluated using the intraclass correlation coefficient (ICC). **Results**: The study population consisted of Gleason score (GS) 6 (*n* = 12), GS 7 (*n* = 34), GS 8 (*n* = 1), and GS 9 (*n* = 3). Significant differences were found for all parameters (*p* < 0.0001). Mean differences were as follows: K^trans^, −0.2102 (95% confidence interval; −0.2687 to −0.1518); k_ep_, −0.7632 (−0.9005 to −0.6258); v_e_, −0.1507 (−0.2422 to −0.05907); v_p_, −0.02929 (−0.03383 to −0.02476). ICCs for average measures were as follows: K^trans^, 0.2989 (−0.2355 to 0.6021); k_ep_, 0.6883 (0.4507 to 0.8231); v_e_, −0.1331 (−0.9967 to 0.3570); v_p_, 0.2653 (−0.3106 to 0.5881). **Conclusion:** All perfusion parameters were significantly different between the two solutions. Therefore, comparison of perfusion parameters across different solutions is not recommended.

## Simple Summary

Dynamic contrast-enhanced magnetic resonance imaging (DCE-MRI) has emerged as a potential surrogate marker in non-invasive evaluation of tumor angiogenesis. Prostate cancer (PCa) is one of the cancers for which treatment is individualized according to tumor aggressiveness. There have been attempts to assess PCa aggressiveness with DCE-MRI using various pharmacokinetic models and diverse post-processing solutions from different vendors. However, the results have been inconsistent and widely variable, requiring further verification. Therefore, the aim of this study was to investigate the inter-vendor variability of perfusion parameters derived from DCE-MRI in patients with PCa. The high variability of perfusion parameters across solutions should be considered when using quantitative perfusion parameters to evaluate treatment response.

## 1. Introduction

Multiparametric magnetic resonance imaging (MRI) plays a central role in the evaluation of prostate cancer (PCa), and dynamic contrast-enhanced MRI (DCE-MRI) is an important component of mpMRI for the assessment of tumor location, extent, biologic behavior, and recurrent disease [1]. PCa typically demonstrates earlier and more intense enhancement than the surrounding normal prostate tissue on DCE-MRI [2]. This finding is thought to reflect tumor angiogenesis, in which increased production of angiogenic factors such as vascular endothelial growth factor leads to the development of abnormal micro-vessels that are more fragile and permeable than normal vessels [1,3,4]. Because DCE-MRI can depict these vascular characteristics of PCa, its role in the evaluation of tumor angiogenesis has been investigated [5,6,7,8].

Despite its potential, DCE-MRI has not yet been fully incorporated into routine clinical practice. Measurements derived from DCE-MRI can be influenced by multiple factors, including hemodynamic conditions, arterial input function (AIF) selection, pharmacokinetic modeling, and post-processing implementation, all of which may affect reproducibility [9]. Although attempts have been made to use DCE-MRI for evaluation of response to anti-angiogenic and anti-vascular therapies, quantitative parameters must first be shown to be reproducible before they can be used reliably as imaging biomarkers [9,10,11]. In addition, variability may arise from differences among vendor-specific solutions used for perfusion analysis. Previous studies have demonstrated inter-vendor variability of DCE-MRI-derived perfusion parameters in uterine leiomyoma and rectal cancer [4,12]. However, to our knowledge, such inter-vendor variability has not been sufficiently investigated in patients with prostate cancer. Therefore, the purpose of this study was to investigate the variability of perfusion parameters derived from DCE-MRI between two commercially available solutions in patients with prostate cancer.

## 2. Materials and Methods

This retrospective study was approved by the institutional review board and informed consent was waived.

### 2.1. Patient Selection Criteria

We retrospectively reviewed the medical records of patients who underwent radical prostatectomy for pathologically confirmed prostate cancer between December 2021 and September 2022. Patients were considered eligible if they had undergone preoperative prostate MRI, including DCE-MRI, at our institution; had available whole-mount pathologic topographic maps; and had DCE-MRI datasets that could be analyzed on both solutions. Cases were excluded when the tumor could not be confidently localized on MRI, when MRI findings could not be matched with the pathologic lesion, when the lesion was too small to allow reliable segmentation, or when image transfer to either solution was unsuccessful.

### 2.2. MRI Acquisition

All examinations were performed using a single 3.0-T MRI scanner (Signa Architect, GE Healthcare, Waukesha, WI, USA) equipped with a body array coil, using the same acquisition protocol and reconstruction settings throughout the study period. The standard prostate MRI protocol included multiplanar T2-weighted imaging, axial diffusion-weighted imaging, pre-contrast T1 mapping, dynamic contrast-enhanced imaging, and axial post-contrast fat-suppressed T1-weighted imaging. Pre-contrast T1 mapping was obtained with a dual–flip angle method (5° and 15°). For dynamic contrast-enhanced imaging, a three-dimensional spoiled gradient-echo sequence was used. The acquisition parameters were repetition time/echo time of 3.7/1.6 msec, flip angle of 8°, field of view of 300 × 300 mm, section thickness of 2 mm, no inter-slice gap, matrix size of 224 × 160, and 32 sections. Dynamic imaging was repeated 60 times with a temporal resolution of 3.6 s, allowing coverage of the entire prostate gland. Contrast material was administered intravenously as gadoterate meglumine (Dotarem, Guerbet, Villepinte, France) at 0.1 mmol/kg (0.2 mL/kg) with an injection rate of 3.0 mL/s, followed by a 30 mL saline flush at the same rate. The full sequence parameters are presented in Table 1.

### 2.3. Measurements of Perfusion Parameters

The DCE-MRI datasets were first organized in the PACS system (M6; INFINITT Healthcare, Seoul, Republic of Korea) and were subsequently transferred to workstations running the two commercially available solutions, MR permeability on IntelliSpace Portal version 10.1 (Philips, The Netherlands) and GenIQ on AW Server 3.2 (GE Healthcare, Waukesha, WI, USA) for pharmacokinetic analysis. Both solutions applied the extended Tofts model, and the population-based AIF was commonly selected for the initial analyses for both solutions. The extended Tofts model is a pharmacokinetic model used to describe the tissue contrast-agent concentration–time curve in DCE-MRI. The model assumes that the contrast agent distributes between the intravascular plasma compartment and the extravascular extracellular space, with the AIF serving as the vascular input [7,13]. Unlike the conventional Tofts model, the extended Tofts model additionally incorporates the plasma volume fraction, thereby accounting for the vascular contribution to tissue enhancement. Accordingly, it allows estimation of K^trans^, k_ep_, v_e_, and v_p_. K^trans^ represents the volume transfer constant from plasma to the extravascular extracellular space, k_ep_ represents the rate constant for contrast reflux from the extravascular extracellular space back to plasma, v_e_ represents the volume fraction of the extravascular extracellular space, and v_p_ represents the plasma volume fraction [7,13]. Specifically, AIF could be selected from three bi-exponential model presets according to the contrast injection duration, categorized as short (<5 s), medium (5–10 s), and long (>10 s) on the MR permeability application in ISP. In the present study, the medium preset was used. For exploratory analysis, individual AIF was applied to observe platform-specific differences in perfusion parameters between the individual and population-based AIF methods. The analyzed parameters included K^trans^, k_ep_, v_e_, and v_p_. K^trans^ reflects the transfer of contrast material from the intravascular plasma compartment into the extravascular extracellular space, whereas k_ep_ represents reflux from the extravascular extracellular space back into the plasma compartment. The fractional volumes of the extravascular extracellular space and plasma were expressed as ve and v_p_, respectively [13].

Two radiologists with 15 and 4 years of experience, respectively, reviewed the T2-weighted images, diffusion-weighted images, and corresponding apparent diffusion coefficient (ADC) maps. Both readers were blinded to the Gleason score of each prostate cancer and assigned the Prostate Imaging-Reporting and Data System (PI-RADS) score by consensus on the basis of PI-RADS version 2.1. The zonal location and tumor border were determined by consensus for each tumor-bearing section, after radiologic-pathologic correlation using the topographic maps. In patients with multiple lesions, the largest lesion was selected as the index tumor. Freehand regions of interest were then manually drawn along the tumor margin on the DCE images by the radiologist with 4 years of experience. Segmentation could not be transferred between the two solutions. Therefore, it was redone separately on both platforms. After segmentation, the perfusion values obtained from the tumor-bearing sections were averaged to derive representative whole-tumor values for each parameter.

### 2.4. Histology

Experienced urologic surgeons performed radical prostatectomy in all study patients. After excision, the prostate specimens were submitted for standard histopathologic processing, including formalin fixation, paraffin embedding, and sequential sectioning from the base to the apex. Hematoxylin and eosin–stained slides were then reviewed by a dedicated pathologist, who established the Gleason score for each prostate cancer lesion [14]. Whole-mount topographic maps obtained from these sections served as the reference standard for tumor segmentation on MRI.

### 2.5. Statistical Analysis

Statistical analysis was performed using MedCalc (MedCalc software version 22.032, Mariakerke, Belgium). For each perfusion parameter, paired measurements obtained from the two solutions were analyzed. Agreement between the two solutions was assessed using Bland–Altman analysis, from which the mean difference and the upper and lower limits of agreement were calculated together with their 95% confidence intervals (CIs). In addition, the null hypothesis that the mean difference between paired measurements is zero was tested for each parameter. Reproducibility between the two solutions was evaluated using the intraclass correlation coefficient (ICC), and 95% CIs were calculated for both single and average measures. ICC values were interpreted as poor (<0.40), fair (0.40–0.59), good (0.60–0.74), and excellent (0.75–1.00) [15]. An inter-observer reproducibility analysis was not performed for the segmentation process itself for the purposes of this study. A *p*-value less than 0.05 was considered to indicate statistical significance.

## 3. Results

### 3.1. Patient Demographics

A total of 50 patients (mean age, 71.6; range, 56–86) with pathologically confirmed prostate cancer were included and analyzed. The mean prostate-specific antigen level was 20.8 ng/mL (range, 0.85–131). Tumor location was the peripheral zone in 23 patients (46%), the transition zone in 19 (38%), and diffuse in 8 (16%). PI-RADS version 2.1 scores were 3 in 4 patients (8%), 4 in 17 (34%), and 5 in 29 (58%). Their demographic information is provided in Table 2.

### 3.2. Inter-Vendor Variability of Perfusion Parameters

**Figure 1:**
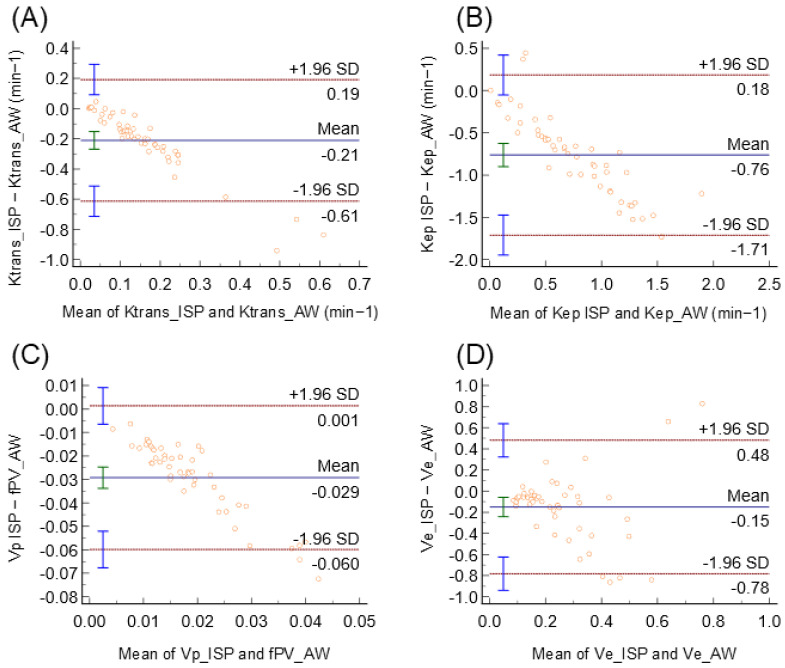
Bland–Altman plots for inter-vendor variability of DCE-MRI perfusion parameters between the two solutions. (**A**) K^trans^; (**B**) k_ep_; (**C**) v_p_; and (**D**) v_e_. For each parameter, the *x*-axis represents the mean of the paired measurements from the two solutions, and the *y*-axis represents the difference between the paired measurements. The solid line indicates the mean difference, and the dashed lines indicate the 95% limits of agreement. Error bars represent the 95% confidence intervals. Wider limits of agreement indicate greater inter-vendor variability. v_e_, extravascular extracellular space volume fraction; K^trans^, volume transfer constant; k_ep_, rate constant; v_p_, fractional volume of plasma.

Bland–Altman analysis revealed significant differences in all four perfusion parameters between the two solutions (Figure 1). The mean differences were −0.2102 (95% CI, −0.2687 to −0.1518) for K^trans^, −0.7632 (95% CI, −0.9005 to −0.6258) for k_ep_, −0.1507 (95% CI, −0.2422 to −0.05907) for v_e_, and −0.02929 (95% CI, −0.03383 to −0.02476) for v_p_. These differences were statistically significant for all parameters (*p* < 0.0001 for K^trans^, k_ep_, and v_p_; *p* = 0.0018 for v_e_). The corresponding limits of agreement ranged from −0.6136 to 0.1931 for K^trans^, −1.7105 to 0.1841 for k_ep_, −0.7822 to 0.4809 for v_e_, and −0.05989 to 0.001304 for v_p_. The detailed results for all of the perfusion parameters are summarized in Table 3.

### 3.3. Reproducibility of Perfusion Parameters Between the Two Solutions

Among the four parameters, k_ep_ showed good reproducibility between the two solutions according to the predefined ICC interpretation criteria, whereas K^trans^, v_e_, and v_p_ showed poor reproducibility. The ICC showed variable reproducibility across the four perfusion parameters. For single measures, the ICCs were 0.1757 for K^trans^, 0.5248 for k_ep_, −0.06239 for v_e_, and 0.1529 for v_p_. For average measures, the corresponding ICCs were 0.2989 (95% CI, −0.2355 to 0.6021) for K^trans^, 0.6883 (95% CI, 0.4507 to 0.8231) for k_ep_, −0.1331 (95% CI, −0.9967 to 0.3570) for v_e_, and 0.2653 (95% CI, −0.3106 to 0.5881) for v_p_. The detailed results are summarized in Table 4.

## 4. Discussion

Our results revealed that all perfusion parameters were significantly different between the two solutions. Only k_ep_ showed good reproducibility, whereas the other perfusion parameters derived from DCE-MRI showed poor reproducibility between the two vendors’ solutions. Our observation corresponds well with previous studies on rectal cancer and uterine myoma [4,12]. Beuzit et al. reported that poor reproducibility was found in the calculated perfusion parameters derived from different vendors’ perfusion analysis solutions in patients with rectal cancer [12]. Specifically, ICCs of K^trans^, k_ep_ and v_e_ ranged from 0.1 and 0.16, which indicated very poor reproducibility. In addition, the within-subject coefficients of variation in K^trans^, k_ep_, and v_e_ were 67%, 53% and 47%, respectively, between the two vendors’ solutions. Those values are markedly beyond the upper limit of agreement of 20%, which was suggested by the Quantitative Imaging Biomarkers Alliance (QIBA) DCE-MRI technical committee. Heye et al. also reported that the ICCs for the analyzed perfusion analysis solutions showed 0.33–0.68 for K^trans^, 0.02–0.81 for k_ep_, and −0.03–0.72 for v_e_, which indicated a very wide range from poor to moderate reproducibility [4]. The within-subject variation across four perfusion analysis solutions, which were not used in our study, was in a wide range of 48–69% for K^trans^, 37–60% for k_ep_, and 28–74% for v_e_ in patients with uterine myoma. In contrast, each vendor’s solution showed promising results in terms of intra-observer and inter-observer agreement. Specifically, Wang et al. observed an excellent intra- and inter-observer reproducibility (>0.9) for K^trans^, k_ep_, and v_e_ and a good reproducibility (0.84–0.89) for v_p_ derived from DCE-MRI in patients with renal cell carcinoma by using the Omni-Kinetics workstation (GE Healthcare) [16]. In addition, Park et al. also reported an excellent inter-observer reproducibility for K^trans^ (0.939, 95% CI, 0.913–0.959) and k_ep_ (0.920, 95% CI, 0.885–0.946) and good reproducibility for v_e_ (0.834, 95% CI, 0.760–0.888) and v_p_ (0.886, 95% CI, 0.823–0.926) in patients with prostate cancer by using ISP version 10 [17]. Those observations reflect that although the variability across vendors’ solutions is beyond the limits of QIBA recommendations, the reproducibility of a single vendor’s solution is acceptable for longitudinal comparison in each individual patient.

**Figure 2:**
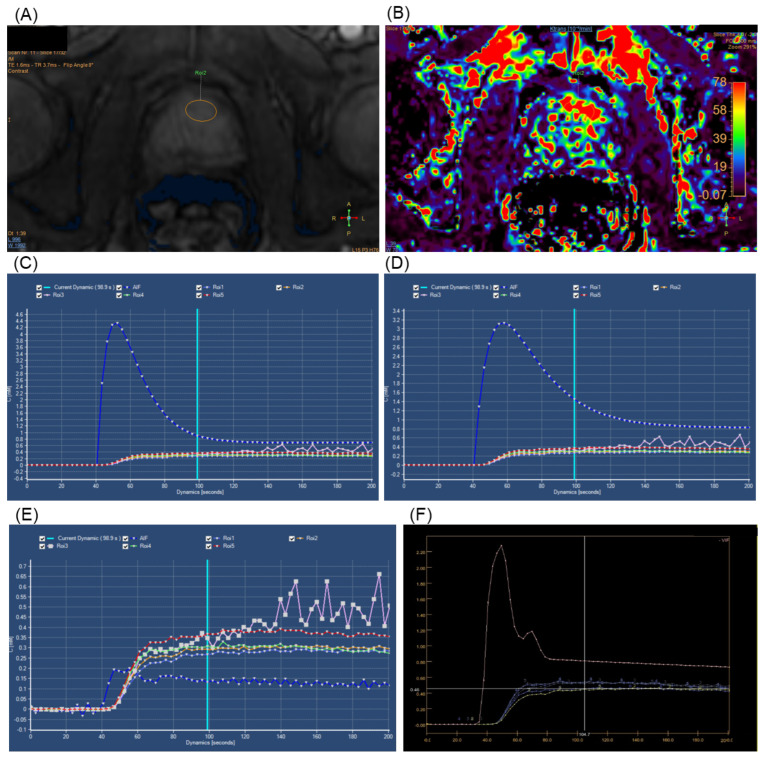
Comparison of arterial input function (AIF) time-concentration curves between solutions (A to E, ISP; F, GenIQ) and presets in a 66-year-old man with prostate cancer in the left middle transition zone (Gleason score 6). Multiple ROIs from tumor are presented in various colors. (**A**) Axial DCE-MRI image on IntelliSpace Portal (ISP) showing placement of a region of interest (ROI) within the index tumor. (**B**) Corresponding K^trans^ map with the tumor ROI overlaid. (**C**) The ISP medium preset AIF, showing a peak arterial concentration of approximately 4.4 mM. The calculated K^trans^ (volume transfer constant) was 0.1/min. (**D**) ISP short preset AIF, showing a lower arterial peak of approximately 3.2 mM; The calculated K^trans^ was 0.1/min. (**E**) Individual AIF in ISP; AIF graph is in blue and tumor concentration graphs from 5 tumor ROIs are in other colors. The average calculated K^trans^ value was 2.8/min (**F**) GenIQ population-based AIF, displayed with absolute concentration on the *y*-axis, with an arterial peak of approximately 2.3 mM. The calculated K^trans^ was 0.48/min. Differences in AIF preset and type are assumed to contribute to variability in estimated perfusion parameters.

**Figure 3:**
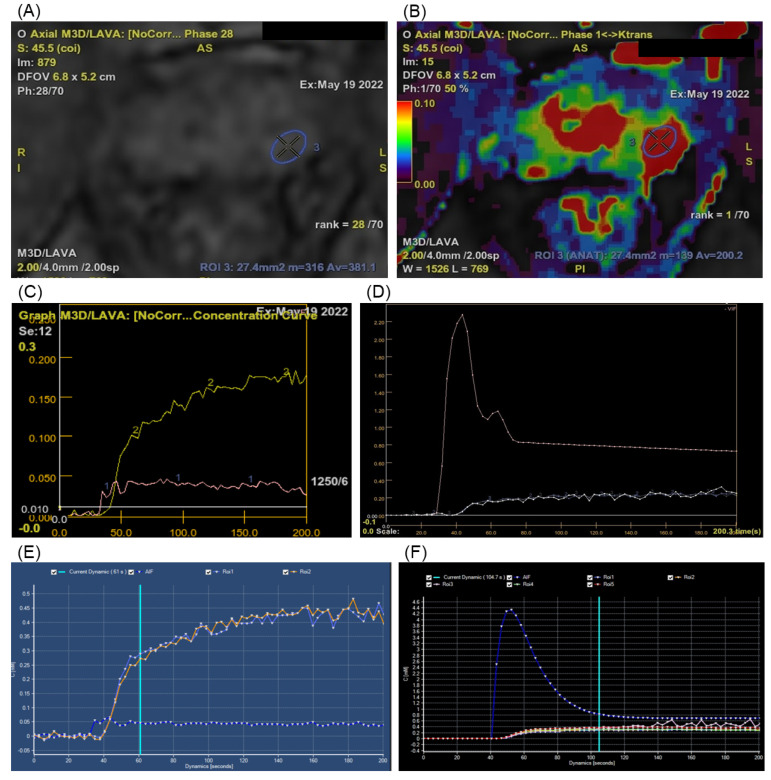
Comparison of arterial input functions (AIF) time-concentration curves obtained from two solutions (A to D, GenIQ; E-F, ISP) in a 70-year-old man with prostate cancer in the left middle peripheral zone (Gleason score 7). Multiple ROIs from tumor are presented in various colors. (**A**) Axial DCE-MRI image on GenIQ showing placement of a region of interest (ROI) within the index tumor. (**B**) Corresponding K^trans^ (volume transfer constant) map with the tumor ROI overlaid. (**C**) Individual AIF in GenIQ, derived from manually placed external iliac arterial ROI; AIF graph is in orange, and tumor graph is in yellow. The calculated K^trans^ was 0.2/min. (**D**) Population-based AIF in GenIQ, with an arterial peak of approximately 2.3 mM. The calculated K^trans^ was 0.2/min. (**E**) Individual AIF in IntelliSpace Portal (ISP); AIF graph is in blue; tumor graphs are from ROI 1 and 2. The average calculated K^trans^ was 10.1/min. (**F**) ISP population-based medium preset AIF, showing arterial peak of approximately 4.4 mM. The calculated K^trans^ was 0.1/min. These differences in AIF type and curve profile may contribute to variability in estimated perfusion parameters.

We explicitly acknowledge that variability may differ across scanners, field strengths, or acquisition protocols, and results may not directly extrapolate to multi-center or multi-vendor acquisition settings. Our findings, together with the existing literature, suggest that inter-vendor variability may be related to differences in solution implementation and parameter fitting across vendors. Differences in population-based AIF presets between the two solutions may be one of the contributors to the high inter-vendor variability of the measured perfusion parameters. Although both solutions used the extended Tofts model, the AIF curves were not identical, and the parameter estimation in this model is highly dependent on the assumed AIF (Figure 2 and Figure 3). Based on our observation, the peak concentration of AIF on the short preset was 3.2 mM, whereas the medium preset showed a higher peak concentration of 4.4 mM. This indicates that the preset selection may influence the estimated K^trans^. Under the same tumor concentration at a fixed time point, a higher AIF concentration yields a lower estimated K^trans^ value. In contrast, the population-based AIF peak concentration in GenIQ was 2.3 mM, which was the lowest. These observed AIF differences may have contributed, at least in part, to the tendency for K^trans^ values obtained with ISP to be lower than those obtained with GenIQ. Our observation is supported by Huang et al., who showed that variation in AIF determination can substantially affect pharmacokinetic estimates in prostate DCE-MRI [18].

These findings have important practical implications. For longitudinal follow-up, quantitative comparison should be performed with the same vendor-specific solution whenever possible. If different vendors are used, numerical changes in perfusion parameters may reflect software-related variation rather than a true biologic change. This point is consistent with current recommendations for reproducibility in quantitative DCE-MRI. The QIBA DCE-MRI Profile was developed specifically to improve standardization, and it suggests that technical variance must be controlled before quantitative changes can be interpreted clinically [10,11].

In our analysis, the difference between population-based and individual AIF-derived parameters appeared to vary between the two solutions. Our results also support that an individual AIF may be preferable to a population-based AIF. Although a population-based AIF is convenient in perfusion analysis, it cannot fully reflect patient-specific hemodynamic factors such as cardiac output, circulation time, or vascular disease [19]. In contrast, an individual AIF is more likely to reflect the actual physiology of the patient. In our observation, the gap between population-based AIF and individual AIF appeared larger in ISP than in GenIQ (Figure 2 and Figure 3). This observation is assumed to be due to the different settings of the AIF curves in the two solutions. As mentioned above, in GenIQ, the population-based AIF showed the lowest peak concentration among the compared models, with a maximum peak of 2.3 mM. In the same solution, the values obtained with the individual AIF were relatively closer to those obtained with the population-based AIF, suggesting less variation according to AIF type. On the other hand, when the individual AIF was applied in ISP, K^trans^ showed a much larger deviation from those obtained with the population-based AIF than in GenIQ. In several cases, this discrepancy was on the order of 10-fold to 100-fold, indicating a markedly high variability. These observations suggest solution-dependent differences depending on AIF type. However, this observation was derived from patients with prostate cancer; thus, it should be interpreted cautiously in other malignancies. We suggest that the variability of the estimated perfusion values can be attributed to the AIF type. In addition, measurement based on individual AIF rather than relying only on a population-based AIF may provide more physiologically relevant pharmacokinetic perfusion parameters in DCE-MRI.

This study has several limitations. First, we compared only two commercial solutions that were available in our institution, although both are widely used. Therefore, our findings may not apply to all DCE-MRI perfusion analysis solutions. Second, though we meticulously searched for the pharmacokinetic model in detail in white papers and users’ manuals, the detailed pharmacokinetic algorithms were not fully accessible, which may limit our ability to identify other causes of the wide inter-vendor variability. Other hidden vendor-specific factors, including fitting techniques, signal-to-concentration conversion, T1 mapping implementation, and parameter limitations, may also have contributed to the observed variability but were not fully accessible. Based on our observations, we consider the AIF type and preset selection to be major potential contributors to high inter-vendor variability. Third, intra- or inter-reader variability was not assessed because this study focused on inter-vendor variability across solutions. Finally, the relatively wide CIs for some parameters may partly be due to the small cohort size and inherently high inter-vendor variability.

## 5. Conclusions

In conclusion, all DCE-MRI-derived perfusion parameters obtained in patients with prostate cancer showed a wide range of inter-vendor variability. This variability is likely related, at least in part, to differences in vendor-specific AIF type and preset. Therefore, we suggest that comparison of perfusion parameters across different solutions is not recommended, and the same vendor-specific solution be used for longitudinal follow-up.

## Figures and Tables

**Table 1:** MRI sequences and scanning parameters.

Parameter	T2WI Axial/Coronal/Sagittal	DWI Axial	Pre-Contrast T1WI LAVA Axial	DCE T1WI LAVA Axial	Post-Contrast FS T1WI Axial
TR (ms)	4680–4930	5260	3.7	3.7	836
TE (ms)	75–100	88.5	1.6	1.6	8.0
Slice thickness (mm)	3	3	2	2	3
Interslice gap (mm)	0.3	0.3	0	0	0.3
Matrix size	400 × 320	120 × 120	224 × 160	224 × 160	340 × 256
Flip angle (degree)	-	-	5/15	8	-
FOV (mm × mm)	220 × 220	240 × 240	300 × 300	300 × 300	220 × 220
b-values (s/mm^2^)	-	0, 100, 1000, 2000	-	-	-
NEX	1	2, 2, 4, 8	0.7	0.7	1
Number of slices	30/30/26	30	32	32	30
Acquisition time (min/s)	1 min 29 s/1 min 44 s/1 min 51 s	7 min 53 s	3 s/3 s	3 min 25 s	2 min 27 s

T1WI, T1-weighted image; T2WI, T2-weighted image; DWI, diffusion-weighted image; FS, fat-saturated; LAVA, 3-dimensional spoiled gradient echo sequence; DCE, dynamic contrast-enhanced; TR, repetition time; TE, echo time; FOV, field of view; NEX, number of excitations.

**Table 2:** Demographic information about enrolled patients.

Parameter	Total	CSC (*n* = 38)	Non-CSC (*n* = 12)
Mean PSA ng/mL [range]	20.8 [0.85–131]	23 [0.85–131]	13.7 [2.15–21.2]
Tumor location, *n* (%)			
Peripheral zone	23 (46)	17	6
Transition zone	19 (38)	13	6
Diffuse	8 (16)	8	0
PI-RADS v2.1, *n* (%)			
3	4 (8)	2	2
4	17 (34)	11	6
5	29 (58)	25	4
ISUP grade group, *n*			
Grade 1 (GS 6)	12		12
Grade 2 (GS 3 + 4)	22	22	
Grade 3 (GS 4 + 3)	12	12	
Grade 4 (GS 4 + 4)	1	1	
Grade 5 (GS 4 + 5)	3	3	

CSC, clinically significant cancer; PI-RADS v2.1, Prostate Imaging Reporting and Data System version 2.1; PSA, prostate-specific antigen; ISUP, International Society of Urologic Pathologists; GS, Gleason score.

**Table 3:** Differences in the measurements of pharmacokinetic parameters of dynamic contrast-enhanced MRI on GenIQ and IntelliSpace Portal.

Values	K^trans^	k_ep_	v_e_	v_p_
Mean	−0.2102	−0.7632	−0.1507	−0.02929
95% CI	−0.2687 to −0.1518	−0.9005 to −0.6258	−0.2422 to −0.05907	−0.03383 to −0.02476
*p*-value	<0.0001	<0.0001	0.0018	<0.0001
Lower limit	−0.6136	−1.7105	−0.7822	−0.05989
95% CI	−0.7142 to −0.5130	−1.9468 to −1.4742	−0.9398 to −0.6247	−0.06769 to −0.05209
Upper limit	0.1931	0.1841	0.4809	0.001304
95% CI	0.09249 to 0.2937	−0.05219 to 0.4205	0.3234 to 0.6385	−0.006497 to 0.009105

Data are mean values. CI, confidence interval; K^trans^, volume transfer constant; k_ep_, rate constant; v_e_, extravascular extracellular space volume fraction; v_p_, fractional volume of plasma.

**Table 4:** Intraclass correlation coefficients for pharmacokinetic parameters measured using GenIQ and IntelliSpace Portal on dynamic contrast-enhanced MRI.

Values	K^trans^	k_ep_	v_e_	v_p_
Single measures	0.1757	0.5248	−0.06239	0.1529
95% CI	−0.1053 to 0.4308	0.2909 to 0.6994	−0.3326 to 0.2173	−0.1344 to 0.4166
Average measures	0.2989	0.6883	−0.1331	0.2653
95% CI	−0.2355 to 0.6021	0.4507 to 0.8231	−0.9967 to 0.3570	−0.3106 to 0.5881

CI, confidence interval; K^trans^, volume transfer constant; k_ep_, rate constant; v_e_, extravascular extracellular space volume fraction; v_p_, fractional volume of plasma.

## Data Availability

The data presented in this study are available on request from the corresponding author. The data are not publicly available due to patients’ privacy.
